# Oxytocin and Vasopressin Receptor Gene Variation as a Proximate Base for Inter- and Intraspecific Behavioral Differences in Bonobos and Chimpanzees

**DOI:** 10.1371/journal.pone.0113364

**Published:** 2014-11-18

**Authors:** Nicky Staes, Jeroen M. G. Stevens, Philippe Helsen, Mia Hillyer, Marisa Korody, Marcel Eens

**Affiliations:** 1 University of Antwerp, Department of Biology, Ethology research group, 2610, Antwerp, Belgium; 2 Centre for Research and Conservation, Royal Zoological Society of Antwerp, 2018, Antwerp, Belgium; 3 San Diego Zoo Institute for Conservation Research, Escondido, CA 92027, United States of America; 4 Molecular Systematics Unit, Western Australian Museum, Perth, WA 6106, Australia; Oregon Health and Science University, United States of America

## Abstract

Recent literature has revealed the importance of variation in neuropeptide receptor gene sequences in the regulation of behavioral phenotypic variation. Here we focus on polymorphisms in the oxytocin receptor gene (*OXTR*) and vasopressin receptor gene 1a (*Avpr1a*) in chimpanzees and bonobos. In humans, a single nucleotide polymorphism (SNP) in the third intron of *OXTR* (rs53576 SNP (A/G)) is linked with social behavior, with the risk allele (A) carriers showing reduced levels of empathy and prosociality. Bonobos and chimpanzees differ in these same traits, therefore we hypothesized that these differences might be reflected in variation at the rs53576 position. We sequenced a 320 bp region surrounding rs53576 but found no indications of this SNP in the genus *Pan*. However, we identified previously unreported SNP variation in the chimpanzee *OXTR* sequence that differs from both humans and bonobos. Humans and bonobos have previously been shown to have a more similar 5′ promoter region of *Avpr1a* when compared to chimpanzees, who are polymorphic for the deletion of ∼360 bp in this region (+/− DupB) which includes a microsatellite (RS3). RS3 has been linked with variation in levels of social bonding, potentially explaining part of the interspecies behavioral differences found in bonobos, chimpanzees and humans. To date, results for bonobos have been based on small sample sizes. Our results confirmed that there is no DupB deletion in bonobos with a sample size comprising approximately 90% of the captive founder population, whereas in chimpanzees the deletion of DupB had the highest frequency. Because of the higher frequency of DupB alleles in our bonobo population, we suggest that the presence of this microsatellite may partly reflect documented differences in levels of sociability found in bonobos and chimpanzees.

## Introduction

Understanding the evolution of behavioral differences in closely related species is one of the major challenges in modern behavioral ecology. Recently, genetics have been increasingly used to understand how these differences occur [1], [2]. For example, differences in genes coding for neuropeptide receptors in the brain have been linked to variation in social behavior between species [3]–[5]. Primate studies, especially on great apes, are particularly interesting to increase our understanding of the evolution of sociality that led to the current complex social structures found in human populations. In terms of human sociality, our two closest living relatives, chimpanzees *(Pan troglodytes* or *P. t.)* and bonobos *(Pan paniscus* or *P. p.)* offer an interesting comparative framework, as they differ in key aspects of social behavior [6]–[8]. The common ancestor of these two sister-species separated from the hominoid line approximately 5 million years ago, and from each other as recently as 0.8 to 2.5 million years ago [9]–[11]. Despite this close phylogenetic relatedness, most research has focused on the dichotomy between these two species [12], [13]. Bonobos are often described as being highly sexual, showing lower levels of aggression and higher levels of reconciliatory tendencies than chimpanzees. Additionally, bonobos are female co-dominated compared to a male dominated society in chimpanzees [12]–[18]. However, other studies now challenge this view and suggest more behavioral continuity between these species for these traits [6]–[8], [19]. The debate on the distinctiveness of these two species reflects recent efforts to determine more proximate causes of interspecies differences and how these evolved in early hominids. Although it is generally accepted that complex social behavior is regulated by a combination of genes, the environment, and an interaction of the two [20], studies have identified candidate genes showing large effects on behavioral variation [1]. Within this study we focus on variation in two genes known to play a role in the regulation of social behavior, namely the receptor genes for oxytocin (OXT) and arginine vasopressin (AVP). OXT and AVP are neuropeptides that are primarily synthesized in the hypothalamus, transported to the posterior pituitary from where they can be stored or released into the systemic circulation, or within synapses in the brain, where they can interact with receptors in different brain regions [21], [22]. For both OXT and AVP, the genes that code for their receptors in the brain have been explicitly linked to variation in behavioral phenotypes in various species ranging from rodents [23], [24] to primates [25], [26], including humans [27]–[29].

The link between oxytocin receptor gene (*OXTR*) variation and behavior has primarily been studied in humans [30]–[32]. One particular single nucleotide polymorphism (SNP) in the third intron of the oxytocin receptor gene (rs53576 A/G) has recently emerged as an interesting candidate for producing behavioral consequences [33]–[36]. In humans, carriers of the rs53576 risk allele (A) show a reduction in social skills, including empathic capacity [5], [34], [35] and prosocial behavior [37]. Although rs53576 is located in a non-coding intronic region, it could still be of functional importance to regulate behavioral variation. For example, intronic SNPs can affect the relative binding affinity of specific splicing regulatory proteins, resulting in different behavioral phenotypes in humans [38]. However, this does not seem to be the case for rs53576 [39]. Another study describes how transcriptional control elements within the third intron of the human OXTR are involved in transcriptional suppression of the gene [40], which led to the hypothesis that SNPs in this region could be regulatory by altering methylation patterns [41]. Although the underlying mechanism of how rs53576 generates behavioral differences remains largely unknown, there is evidence for structural neural alterations in key oxytocinergic regions [5], [42]. Individuals carrying at least one risk allele (A) show a significant decrease in hypothalamus gray matter, an increased volume of the right amygdala grey matter in males, an increased structural correlation of the hypothalamus and dorsal anterior cingulate cortex (dACG), and an increase in structural coupling of hypothalamus and amygdala [5], [42]. Interestingly, bonobos and chimpanzees show interspecies differences in volume of gray matter of these particular brain regions, and in associated white matter pathways connecting these regions [36]. Compared to chimpanzees, bonobos have a larger hypothalamus and right dorsal amygdala and a larger pathway connecting the amygdala with the ventral anterior cingulate cortex (vACG) [43]. These differences in brain anatomy are thought to be linked with observed interspecies differences in behavior and temperament, including empathy and prosociality [43]. Bonobos are found to perform better at solving tasks related to theory of mind or understanding social causality, which indicates higher empathic sensitivity [44]. Furthermore, in bonobos, higher levels of social tolerance [45] (but see [46]), prosociality [47], [48] and lower levels of inter- and intragroup aggression [8], [13], [49] have been described compared to chimpanzees. In light of these findings, we hypothesized that if bonobos and chimpanzees share the same genetic variation of rs53576 (A/G) in the *OXTR* as found in humans, they might differ in frequencies of these alleles on a species level. This variation could explain differences found in social behavior and organization between these species.

A second candidate gene involved in regulation of social behavior, is the gene coding for the vasopressin receptor 1a (*Avpr1a*). The length of the promoter region of this gene correlates with increased sociability in a variety of mammalian species, including humans [25], . The underlying mechanism has been studied using homologous recombination techniques in rodents. By incorporating the 5′ promoter region of *Avpr1a* from vole species into the *Avpr1a* gene of mice, Donaldson and colleagues [52] have shown that the length of this promoter region partly determines vasopressin receptor 1a distribution patterns in the brain. Use of transcription predictor assays suggests this might be due to alterations of gene expression in these different brain regions [53]. As the length of this promoter region is highly variable, differences in receptor distribution patterns in the brain are found within [50], [54], [55], but also between different vole species [56]. Studies on the social effects related to these different brain receptor patterns, indicated that individuals with longer alleles showed increased levels of social behavior, specifically in parental care and a more definitive partner preference [50], [54]. Based on the results in rodents, it was later suggested that the effect of variation in this specific gene might affect a wider variety of species, including primates [50].

Based on a small sample of two bonobos, Hammock and Young [50] described how humans and bonobos have a more similar 5′ *Avpr1a* promoter region when compared to chimpanzees. The former have three microsatellites in this region named STR1, RS3 and RS1 whereas chimpanzees are polymorphic for the presence of an approximately 360 bp deletion, called the DupB region, which includes the RS3 microsatellite [57]. Although both RS1 and RS3 have been subject of behavioral association studies, the majority of behavioral correlates have been attributed to variation in RS3 [27], [51], [58], [59]. In humans, more RS3 microsatellite repeats correlate with increased prosocial behavior [27] and stronger amygdala activation to face recognition [59]. Furthermore, individuals with longer RS3 alleles show higher promoter activity [60] and have higher *Avpr1a* messenger RNA levels in their hippocampus, where vasopressin is produced, suggesting that this microsatellite plays a role in gene regulation in humans [27]. However, in non-human primates very little is known about the link of RS3 with behavior. Rosso *et al.* [61] found no direct link between the presence of RS3 and male mating behavior across 12 Old World primates. They showed that species with dissimilar male behaviors (e.g. bonobos, orangutans and lar/symphalangus gibbons) all share the presence of the RS3 microsatellite.

Since *Avpr1a* has been linked to intersexual bonding in voles [24], [63] and humans [29], it will be interesting to evaluate if differences in bonding patterns and sociability between closely related *Pan* species covary with the length of the RS3 alleles. Bonobos and chimpanzees are reported to differ in levels of intra- and intersexual bonding. In bonobos, there is evidence for strong male-female association and grooming in all groups [62], [63]. These associations are believed to reflect reproductive efforts [64], while sexual coercion has never been observed [65]. In chimpanzees, bond strength is typified by the strongest bonds occurring between the males, the weakest between females, with male-female bond strength falling intermediary [66]. However, recent evidence shows a more complex picture, with some authors suggesting that bonding patterns in Western chimpanzees more closely resemble the bonobo pattern of intersexual bonding [67]–[69], but Eastern populations show variation in the strength of intra- and intersexual social bonds [70]–[73].

Until now, data on RS3 in bonobos have been based on small sample sizes totaling five individuals [50], [57], [61]. All three studies showed that bonobos do not have the deletion of the DupB region, but no further results on RS3 variation have been published. In chimpanzees, more studies are available that have examined the variation of the RS3 genotype and its relation to behavior. These studies have mainly focused on the deletion of DupB ( = DupB−), which includes the RS3 microsatellite [25], [57], [61], [74], [75]. For Western chimpanzees, the presence of DupB ( = DupB+) has been associated with a variety of social traits and capacities. DupB+/− and DupB+/+ individuals appear to have a “smarter” social behavioral style compared to DupB−/− individuals, meaning they score higher on the number of coalitions, on the amount of grooming received and on likeliness to initiate play [74]. DupB+ males show more affiliative behavior [74], have a more dominant and conscientious personality profile [25], higher scores on a social cognition task, and more responsiveness to socio-communicative cues [75] than DupB−/− males. Interestingly, the allele frequencies for this deletion differ between subspecies of chimpanzees, with a higher prevalence of the deletion occurring in chimpanzees of West-African origin (*P. t. verus*) (DupB- frequency = 0.74 [57] and 0.77 [74]) compared to the Eastern chimpanzees (*P.t. schweinfurthii*) (DupB- frequency = 0.38 [74]). No deletions have been reported for the Central chimpanzees (*P. t. troglodytes*), however the sample size for this subspecies was limited to two individuals [61].

While studies have looked into ecological differences to explain different patterns of intersexual bonding between bonobos, Western and Eastern chimpanzees, it is also possible that variation in long-term association, and perhaps affiliation could partly be due to differences in DupB- allele frequencies. Since few studies have described the variation in *Avpr1a* alleles in bonobos and chimpanzees, especially of Eastern origin, our goal was to investigate the presence of DupB in a larger sample size of bonobos, and describe and compare the RS3 length variation in both bonobos and chimpanzees.

## Materials and Methods

### Samples

Hair or blood samples were obtained from the Centre for Research and Conservation at the Royal Zoological Society of Antwerp, Belgium (bonobo N = 21; chimpanzee N = 8); the Biomedical Primate Research Centre Rijswijk, Netherlands (chimpanzee N = 14); Arnhem Zoo, the Netherlands (chimpanzee N = 7); Chester Zoo, United Kingdom (chimpanzee N = 5) and Beekse Bergen, the Netherlands (chimpanzee N = 1). The San Diego Zoo Institute for Conservation Research (California, United States) PCR amplified DNA that was banked in their Frozen Zoo from 14 bonobos for analysis in their genetics lab or by Macrogen Inc., Korea.

We selected unrelated individuals for both species in order to capture as much variability as possible in the captive populations. For bonobos, we were able to utilize DNA/data from a total of 33 wild-born individuals (founders) and two unrelated F1- offspring of missing founder couples unrelated to each other or the other founders, thereby capturing approximately 90% of the total captive founder variation. Relatedness among these founders was assumed to be very low or non-existent and individuals were captured over a period of more than 30 years from different populations located across the entire bonobo distribution region [76].

For chimpanzees, we collected DNA from a total of 20 wild-born individuals, six individuals of unknown origin and nine unrelated F1-offspring. Subspecies status has been assigned to 32 out of 35 of our chimpanzees [77]. Our sample group consists of 21 *P. t. verus*, two *P. t. schweinfurthii*, one *P. t. troglodytes*, one labeled as *P.t.troglodytes* or *P.t.schweinfurthii* and seven subspecific hybrids primarily *troglodytes x verus*. We extracted DNA using a Puregene Core Kit B (QIAGEN). Human DNA from the main investigators and negative control samples were included in all procedures to rule out contamination during analysis. For both *OXTR* and *Avpr1a* genotyping, we re-analyzed approximately 20% of the samples at least once. Additionally, offspring of the founders were genotyped and used in conjunction with studbook information to validate inheritance patterns of the alleles in this study.

### OXTR

Amplification of the OXTR region surrounding rs53576 in humans was completed for 27 bonobos and 35 chimpanzees (Tables S1 and S3) using the primer set from Wu *et al*. [33]: forward 5′- GCC CAC CAT GCT CTC CAC ATC-3′ and reverse 5′- GCT GGA CTC AGG AGG AAT AGG GAC-3′. Each 25 µL PCR reaction mix contained 1X QIAGEN *Taq* Buffer advanced, 1 mM MgCl_2_, 200 µM dNTP’s, 1.25U 5 PRIME *Taq* DNA Polymerase (5 U/µL), 0.5 µM of both primers and approximately 45 ng genomic DNA. PCR started with an initial incubation at 95°C (5 minutes), followed by 40 cycles at 95°C (30 s), 62°C (40 s), 72°C (40 s) and a final extension period of 10 minutes at 72°C. Samples showing multiple PCR bands were gel extracted based on size selection prior to sequencing. Sequencing of the region of interest was performed by Macrogen Europe (Netherlands). Resultant sequences were aligned to human reference sequences using Geneious (Version 5.5). Human variation within this *OXTR* region was identified using the Ensembl dbSNP database. Potential functional consequences of the observed genetic variation were evaluated in terms of differential binding preference of transcription factors with both the TFSEARCH tool (with a cutoff score of 0.85) [78] and JAPSAR 2014 (with a profile threshold score of 0.75) [79]. We used pedigree analysis to determine SNP variation in an additional 7 bonobo founders by genotyping in total, 16 of their descendants (Table S2).

### Avpr1a

Amplification of the region surrounding the RS3 microsatellite was done for 35 bonobos and chimpanzees, using different primer sets for both species. For bonobos we used the primer set from Bachner-Melman *et al.* [51]: forward 5′-CCT GTA GAG ATG TAA GTG CT-3′ and reverse 5′-TCT GGA AGA GAC TTA GAT GG-3′. As chimpanzees are polymorphic for an ∼360 bp deletion including the reverse bonobo primer region, we developed a new primer set: forward 5′- TTT TTC AGA GGG ATC CTG-3′ and reverse 5′-GGA ATG AGT TAA CAA ATG TTG TAG-3′. Forward primers were fluorescently labeled (6-FAM). The 25 µL PCR reaction mixes contained the same components used for the OXTR reactions except the MgCl_2_ was omitted. PCR cycle conditions were comparable to the OXTR conditions except for the annealing temperature and the number of cycles, which were set to 54°C and 35 cycles respectively. Individuals were genotyped using automated capillary electrophoresis (Macrogen Inc., Korea or ABI 3130 Genetic Analyzer, Life Technologies). For chimpanzees, RS3 amplicons were first visualized on an agarose gel (1.8%). DupB−/− individuals were excluded from further genotyping since they lack the variable RS3 microsatellite region.

### Ethics Statement

No animals were sacrificed or sedated for the purpose of this study. All European DNA samples were provided from existing DNA databanks that collect and store samples following BIAZA guidelines that state that some material may be obtained opportunistically during health checks or other recognized husbandry procedures. Most of these samples were hair samples that were collected non-invasively. In case of blood samples, we followed the BIAZA guidelines that state that no more than 10% of samples taken for veterinary purposes can be used for secondary research purpose. Samples from San Diego Zoo animals were collected opportunistically during routine veterinary checks and approved by the SDZG IACUC (assurance# 12–023). Additional samples from other USA zoos were also collected opportunistically at AZA accredited facilities for population management purposes and are not subject to IACUC approval. Human DNA from the main investigators (NS and JMGS) was acquired non-invasively by use of buccal swabs. As the samples were collected non-invasively and only for the purpose of methodological validation, with no intent to interpret or publish further results regarding these samples, the Scientific Advisory Board of the Royal Zoological Society of Antwerp waived the requirement for human subjects approval for human tissue collection and use in this study. This research was approved by the University of Antwerp (Belgium) and endorsed by the European Breeding Program for bonobos.

## Results

### *OXTR* variation

**Figure 1:**
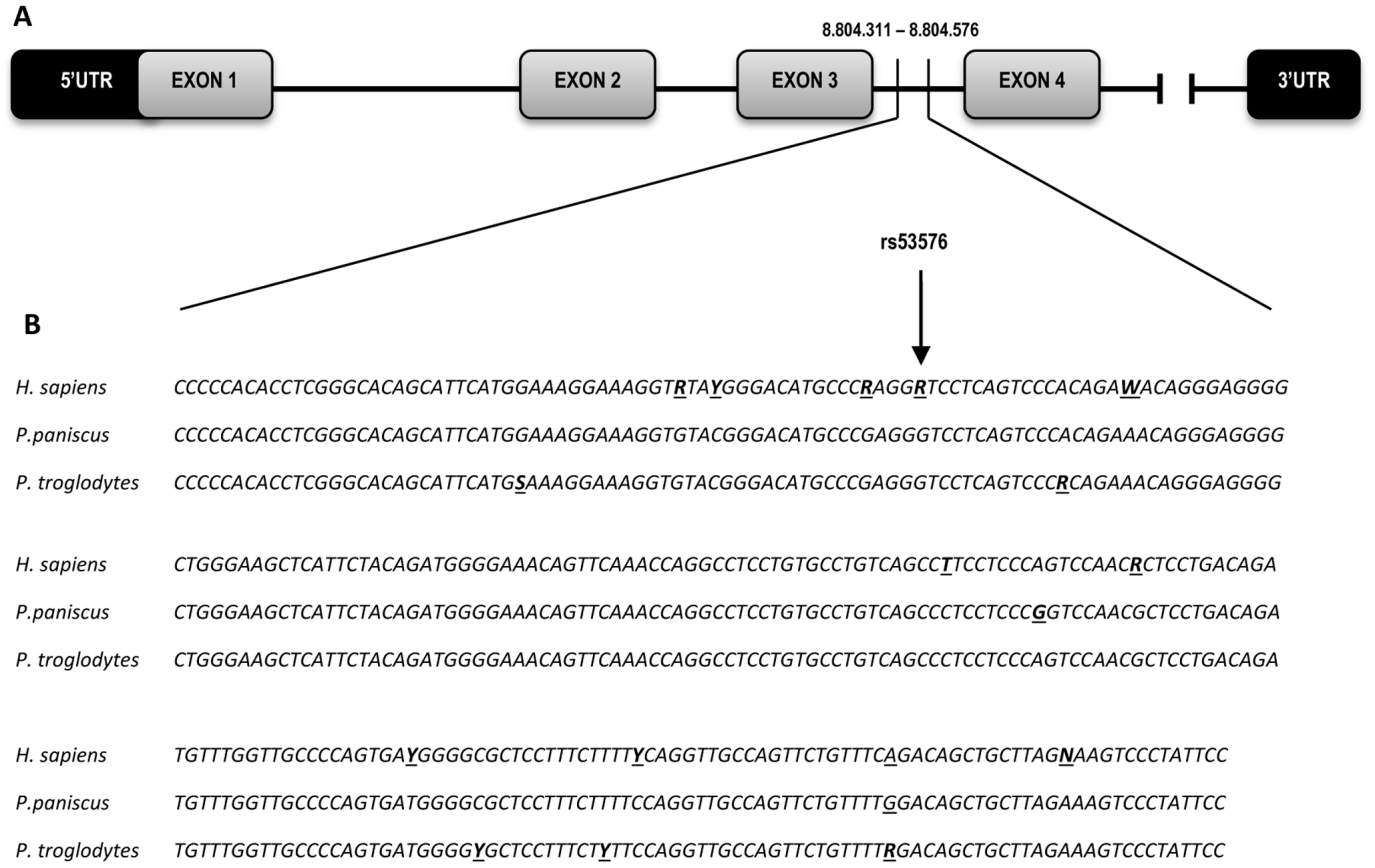
Human, chimpanzee and bonobo oxytocin receptor gene sequences surrounding rs53576 (Chr 3; NC_000003.11). Schematic (A) shows the position of the sequence in the third intron of the human *OXTR* (GRCh37.p13 gi|224589815). Inter-and intraspecific variation is shown in (B). Bold letters indicate single nucleotide polymorphisms indicated following IUPAC nucleotide codes. Underscores indicate positions of interspecific variation. (Human variation was identified using Ensembl dbSNP database.).

We sequenced a 320 bp region surrounding the rs53576 SNP found in humans (reference NCBI: NC_000003.11:g.8804371A>G) in 35 chimpanzees, 27 unrelated bonobos, and an additional 16 descendants of 7 bonobo founders that were missing (Figure 1 and Tables S1–S3). Both species showed no variation at this position, with all individuals being G/G. Further inter-and intraspecific variation is shown in Figure 1. The bonobo dataset showed no SNPs in the sampled region, but we did identify five new SNPs in chimpanzees that are not present in humans or bonobos (Table 1). Four of these SNPs occur at a low frequency, with minor alleles present in one to three individuals, most often in subspecific hybrids of unknown subspecies mixtures. The fourth SNP (ID: NC_006490.3:g.8947139T>C, ss1388116472) on the contrary, is present in a higher proportion, with the minor allele (C) occurring exclusively in the Western subspecies (*P. t. verus*) at a frequency of 0.11. Genotype frequencies for this SNP do not deviate from Hardy-Weinberg equilibrium in our sampled population (χ^2^ = 0.18, df = 1, p = 0.36). Analysis on the presence of regulatory motifs indicate that this SNP overlaps with an SRY transcription factor binding site for the common T allele whereas the “rare” C allele misses this SRY recognition site at the 85 and 75 thresholds in TFSEARCH and JASPAR (2014) respectively. Humans and bonobos, both homozygous T at this position, show no variation in SRY binding at this position. As variation in this SNP is balanced across the age and sex spectrum in chimpanzees, we expect neither of these to influence allele frequencies in our populations (Table S3).

**Table 1:** Position and frequency of alleles and genotypes of SNPs identified in chimpanzee *OXTR* rs53576 surrounding region for a total of 35 unrelated individuals.

SNP ID^1^	NCBI ss number	Allele frequency	Genotype frequency
NC_006490.3:g.8946985G>C	ss1388116469	0.943 (G); 0.057 (C)	0.914 (GG); 0.057 (GC); 0.029 (CC)
NC_006490.3:g.8946997A>G	ss1388116470	0.971 (A); 0.029 (G)	0.971 (AA); 0.029 (GG)
NC_006490.3:g.8947128C>T	ss1388116471	0.971 (C); 0.029 (T)	0.943 (CC); 0.057 (CT)
NC_006490.3:g.8947139T>C	ss1388116472	0.886 (T); 0.114 (C)	0.800 (TT); 0.171 (CT); 0.029 (CC)
NC_006490.3:g.8947163G>A	ss1388116473	0.986 (G); 0.014 (A)	0.971 (GG); 0.029 (AG)

1 ID is based on position of SNP in chimpanzee genome version 74.214 (CHIMP2.1.4 - Chr3).

### *Avpr1a* variation

The RS3 microsatellite was present in all 35 unrelated bonobos, and 11 different alleles were distinguished with length ranging from 463 to 489 bp (Table 2). The most prevalent alleles had a frequency of 0.23 (allele 481) and 0.26 (allele 485). In chimpanzees 11 different RS3 alleles were found, of which two are chimpanzee specific, with length varying from 463 to 494 bp (Table 2). However, the deletion of DupB, including RS3, was present in the majority of chimpanzees (61%, see Table 2).

**Table 2:** Frequency and percentage of RS3 alleles found in an unrelated set of bonobos (*Pan paniscus*) (N = 35) and chimpanzees (*Pan troglodytes*) (N = 35).

	*Pan paniscus*		*Pan troglodytes*	
Allele (bp)	Frequency	Percentage (%)	Frequency	Percentage (%)
140^a^	0	0	43	61
463	4	6	1	1
465	7	10	0	0
469	1	1	0	0
475	0	0	1	1
477	4	6	3	4
479	8	11	2	3
481	16	23	5	7
483	1	1	6	9
484	9	13	0	0
485	18	26	2	3
487	1	1	1	1
489	1	1	1	1
492	0	0	4	6
494	0	0	1	1

a = DupB- allele.

**Figure 2:**
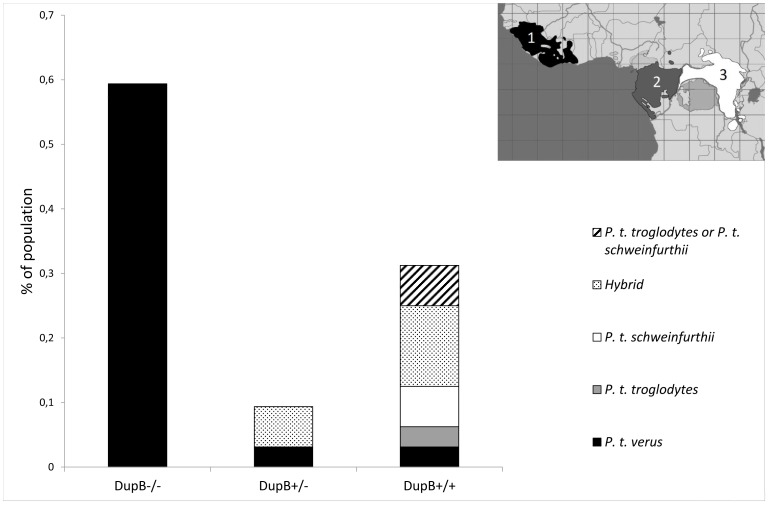
Distribution of DupB genotype frequencies for chimpanzee subspecies. X-axis shows individuals homozygous for the deletion (DupB−/−), heterozygous for the deletion (DupB+/−) or homozygous for the presence of RS3 (DupB+/+). Different chimpanzee subspecies are indicated on the map in black (1) (*Pan troglodytes verus*), dark grey (2) (*Pan troglodytes troglodytes*) and white (3) (*Pan troglodytes schweinfurthii*). Hybrids are not shown on the map. (Map adapted from Prüfer et al 2012) [96].

Subspecies status has been assigned to 32 out of 35 of our wild-born chimpanzees [77]. In our sample, all individuals homozygous for the deletion of DupB (including the RS3 microsatellite) were West-African *P. t. verus* (Figure 2). The individuals belonging to *P. t. troglodytes* and *P. t. schweinfurthii* subspecies were all DupB+/+. We do not expect age or sex to influence our results, as the allelic frequency distribution for both species includes individuals of both sexes and a variety of ages (Tables S1–S3).

## Discussion

We investigated variation in oxytocin (rs53576) and vasopressin (RS3) receptor genes within chimpanzees and bonobos, and their putative relationship to differences in social behavior for both species. As such, we identified previously unreported variation in the oxytocin receptor gene for chimpanzees with possible functional importance but no variation was found in this region for bonobos. For the vasopressin receptor gene, we confirm that there is no deletion of the DupB region, including the RS3 microsatellite, in a large sample of bonobos, whereas in chimpanzees the deletion occurred in most of the sampled individuals.

For the oxytocin receptor gene, the rs53576 position was fixed in both chimpanzees and bonobos. Given a lack of available data on the presence of rs53576 in other ape species, it is unclear whether the variation evident at this site in humans has emerged after divergence from a Panin ancestor, or conversely, whether past variation in the genus *Pan* has simply become fixed. Regardless, given the lack of variation at the rs53576 site in bonobos and chimpanzees, we can conclude that reported interspecies differences relating to empathic sensitivity and prosociality are not driven by variation at this particular site. This suggests that rs53576 only serves functional importance in humans. Recent studies have reported additional SNPs in the human OXTR gene, linked with empathy [28] and autism [33], [80], indicating that it is a more complex interaction affecting behavioral differences and that it is still unclear which precise mechanisms are involved. Accordingly, we assessed bonobos and chimpanzees for additional variation in the region surrounding rs53576. Bonobos showed no further variation, however five additional SNPs were identified within this region in chimpanzees, which is in line with observed variability in human studies [28], [33]–[35]. Further work using quantitative behavioral data is now needed to determine whether these SNPs are of any functional importance in chimpanzees. The question then remains whether they only explain intra- or also interspecific behavioral differences.

Since behavioral association studies in chimpanzees in general have relatively small sample sizes, ss1388116472is the best candidate for future research, as this SNP has a minor allele frequency of more than 5%. Currently there is little information available on the effects of oxytocin in great apes. The few studies done in chimpanzees have reported the importance of oxytocin in social bonding [80], [81]. As bonobos and humans are all TT at this position, this SNP could partly explain reported interspecies differences in social bonding [6], [12], but quantitative comparative data on differences in behavior between the species are needed to confirm this hypothesis. From a functional perspective, it would be interesting to evaluate whether the reduced binding affinity of SRY in the third intron of oxytocin, caused by the “rare” C allele in the Western chimpanzee subspecies (*P. t. verus*), affects the transcription of the oxytocin receptor, and as such the social behavior. More specifically, since SRY transcription factors are exclusively expressed in males, we hypothesize that this SNP should be regarded as a candidate to explain sex-specific differences in oxytocin receptor expression patterns, and therefore sex-specific differences in chimpanzee behavior. Altogether these results underline the importance of species, or even population specific, genetic scans in the upstream flanking regions of regulating genes to fully appreciate the role of silent genetic variation in observed behavioral patterns.

Our second goal was to investigate whether the absence of DupB found in a majority of Western chimpanzees [50], [57], [61] would be found in bonobos using a larger sample size than in previous studies. Our results confirm that all bonobos investigated retain the DupB region containing the microsatellite RS3. They show variation in the length of RS3, with 11 different alleles occurring in our sampled captive population. As found in previous studies [57], [61], our sampled *c*himpanzee population showed a high prevalence (61%) of the DupB- allele. These results suggest that the deletion of the DupB region is chimpanzee specific, and that it is very likely to have occurred after these two sister-species separated from each other. When chimpanzees have RS3 (39%), they show variation in length comparable to what is found in bonobos. Comparing all the sampled chimpanzee subspecies, the DupB deletion occurs only in *P. t. verus* or hybrids of these Western chimpanzees. Although our samples contained relatively few Eastern and Central chimpanzees, these results are in line with previous findings on subspecies differences for the occurrence of the deletion. The frequency of the deletion is found to be rather high in Western chimpanzees [25],[57],[74] while it is the minor allele in Eastern chimpanzees [74]. Furthermore, while the few Eastern and Central chimpanzees we tested makes it difficult to compare RS3 diversity between bonobos and chimpanzee subspecies, our sampled set of bonobos shows RS3 length variation comparable to chimpanzees in general. Due to the high frequency of the DupB- allele in chimpanzees, we have a much higher frequency of DupB+ alleles including RS3 in our bonobo population. As RS3 is known for its role in the regulation of bonding and sociability in voles [50] and humans [29], and deletion of DupB has been associated with a reduction in sociability and affiliation in primarily male Western chimpanzees [26], the variation in DupB might partly reflect differences in levels of sociability and intersexual bonding documented between bonobos and chimpanzees [68]–[73], [82], [83]. Further studies relating sociability to the DupB deletion in chimpanzees are needed to validate the hypothesis regarding its putative role in social bonding differences in bonobos and chimpanzees.

Traditionally chimpanzees are seen as male-male bonded [66], [84], while female-female bonds prevail in bonobos [12]. However this dichotomy has become more blurred as intraspecific variation in bonding patterns was identified in chimpanzees [67], [85], and to some degree in bonobos [82], [86]. Comparisons are hampered as different measures of social bonding are used (e.g. territory overlap, association patterns, proximity, affiliative behavior such as grooming and coalitionary support), while these may not always be equivalent [70], [87]. Finally, short term bonds and long term bonds may differ in functionality [88], but so far few comparable data sets exist on the stability of such bonds. The current evidence suggests that, at least qualitatively, there seems to be more inter-and intraspecific overlap in intersexual bonding than previously recognized [6]. For example, differences between and within Western and Eastern chimpanzees [68], [87], [89] are less clear cut than previously thought [72], [90]. There seems to be variation within the Eastern subspecies, suggesting influences of demography and/or ecological variation, perhaps yet undetected among Western chimpanzees and bonobos, for which fewer long-term field sites exist. Therefore considering differences at species or subspecies level may be less useful than at population level [91]. Quantitative comparisons, to measure and compare strength and stability of bonding between different chimpanzee populations, are not yet available, as methodologies often differ. More integrative measures of components of relationship quality, as have been used for captive chimpanzees [92], [93], can be used to make such quantitative comparisons in the future.

As allele and genotype frequencies for vasopressin show differences between chimpanzee subspecies [74], the question remains to what extent variation of RS3 is responsible for reported subspecies differences in social structure and behavior [8], [94], or whether demographic and/or ecological conditions such as resource supplies can explain observed variation [68], [95]. So far the effect of the deletion of DupB has only been studied within groups of captive Western chimpanzees, so the question whether the behavioral effects of DupB deletion are similar in Eastern and Central chimpanzees remains to be answered. In our current study, Eastern chimpanzees show higher frequencies of DupB+ alleles, and as these longer alleles are in general linked to an increase in male social behavior [26], [54], we might expect Eastern chimpanzee males to show increased levels of sociability and intersexual social bonding compared to Western chimpanzees, which is currently not found by studies in the wild. On the contrary, West African chimpanzees, with fewer DupB+ alleles show stronger intersexual bonding and are therefore suggested to more resemble levels found in bonobos [67]. However, as no quantitative comparisons of intersexual bonding between bonobos and Western chimpanzees have been made so far, it is unclear to what extent bonobo intersexual bonding and sociability really resembles what is found in Western chimpanzees. In general, bonobo parties are still larger than those of both Western and Eastern chimpanzees, and unlike chimpanzees, bonobos are almost never seen solitary in the wild [6]. These differences in party size, combined with reported higher levels of social tolerance and peaceful intergroup interactions, might partly be caused by the fact that the DupB deletion does not occur in bonobos.

The fact that intersexual bonding is higher in Western chimpanzees compared to Eastern chimpanzees, may suggest that ecological factors play a more important role in shaping intersexual association in different subspecies of chimpanzees. Chimpanzees in eastern sites experience higher seasonality compared to Western chimpanzees and bonobos, who have few dry months per year [8]. This results in spatial and temporal variation in food availability and periods of food scarcity in Eastern chimpanzees, which has been shown to influence female social association patterns [8], [67], [96]. We suggest that whereas ecology is an important predictor for subspecies differences in social association, DupB genotypes might be a good predictor for population rather than subspecies differences in sociability in chimpanzees, as populations of the same subspecies may differ in the occurrence of these alleles through mechanisms of genetic drift or natural selection. To address the functionality of this deletion in different (sub)species, comparative data on social behavior in individuals belonging to all three taxa are needed. As studies in chimpanzees have only focused on the deletion of DupB, there is a need for future studies to focus on intra-and interspecific variation in RS3 length in relation to behavior if we want to gain better insight on the exact functional consequences of RS3.

## Supporting Information

Table S1 **Individual information on age, sex, origin and genotype for bonobo samples used in this study.**
(DOCX)

Table S2 **Individual information on age, sex and origin for additional bonobo samples used to identify potential** ***OXTR*** **SNP variation of 7 missing founders.**
(DOCX)

Table S3 **Individual information on age, sex, origin and genotype for chimpanzee samples used in this study.**
(DOCX)
