# Supplementary material for: Oxytocin and Vasopressin Receptor Gene Variation as a Proximate Base for Inter- and Intraspecific Behavioral Differences in Bonobos and Chimpanzees
Source: PLoS One. 2014 Nov 18;9(11):e113364. doi: 10.1371/journal.pone.0113364 (PMC4236177; doi:10.1371/journal.pone.0113364)
Supplement: Table S1 — Individual information on age, sex, origin and genotype for bonobo samples used in this study. (DOCX) [file pone.0113364.s001.docx]

**Table S1. Individual information on age, sex, origin and genotype for bonobo samples used in this study.**

| Studbook number | Sex | Date of Birth | Sire | Dam | Genotype RS3 | Genotyped *OXTR* |
| --- | --- | --- | --- | --- | --- | --- |
| 86 | Female | 1976 | Wild | Wild | 465-479 | x |
| 55 | Female | 1966 | Wild | Wild | 463-465 | x |
| 313 | Female | 1995 | Wild | Wild | 485-485 |  |
| 220 | Female | 1993 | Wild | Wild | 481-485 | x |
| 42 | Female | 1968 | Camillo | Margrit | 469-485 |  |
| 67 | Female | 1971 | Wild | Wild | 485-487 | x |
| 88 | Female | 1978 | Wild | Wild | 481-485 |  |
| 91 | Female | 1978 | Wild | Wild | 484-484 |  |
| 56 | Female | 1966 | Wild | Wild | 481-485 |  |
| 166 | Female | 1982 | Wild | Wild | 479-485 | x |
| 260 | Female | 1999 | Hani | Molaso | 481-485 | x |
| 116 | Female | 1982 | Wild | Wild | 481-485 | x |
| 23 | Female | 1958 | Wild | Wild | 465-489 | x |
| 60 | Female | 1975 | Wild | Wild | 463-465 | x |
| 62 | Female | 1970 | Wild | Wild | 463-485 |  |
| 46 | Female | 1964 | Wild | Wild | 477-481 | x |
| 115 | Female | 1982 | Wild | Wild | 481-485 | x |
| 1009 | Female | 1990 | Wild | Wild | 477-477 | x |
| 102 | Male | 1977 | Wild | Wild | 484-484 |  |
| 64 | Male | 1975 | Wild | Wild | 481-485 |  |
| 97 | Male | 1979 | Wild | Wild | 465-481 |  |
| 169 | Male | 1979 | Wild | Wild | 465-479 |  |
| 57 | Male | 1970 | Wild | Wild | 485-485 |  |
| 104 | Male | 1979 | Wild | Wild | 479-481 |  |
| 34 | Male | 1960 | Wild | Wild | 465-479 | x |
| 69 | Male | 1976 | Wild | Wild | 479-481 | x |
| 68 | Male | 1976 | Wild | Wild | 479-481 | x |
| 54 | Male | 1980 | Wild | Wild | 481-483 | x |
| 167 | Male | 1980 | Wild | Wild | 484-484 | x |
| 1003 | Male | 1985 | Wild | Wild | 481-485 | x |
| 165 | Male | 1978 | Wild | Wild | 463-485 | x |
| 105 | Male | 1979 | Wild | Wild | 479-481 | x |
| 58 | Male | 1970 | Wild | Wild | 484-485 | x |
| 123 | Male | 1981 | Wild | Wild | 477-481 |  |
| 168 | Male | 1980 | Wild | Wild | 484-484 | x |
| 31 | Female | 1951 | Wild | Wild |  | x |
