# Supplementary material for: Oxytocin and Vasopressin Receptor Gene Variation as a Proximate Base for Inter- and Intraspecific Behavioral Differences in Bonobos and Chimpanzees
Source: PLoS One. 2014 Nov 18;9(11):e113364. doi: 10.1371/journal.pone.0113364 (PMC4236177; doi:10.1371/journal.pone.0113364)
Supplement: Table S2 — Individual information on age, sex and origin for additional bonobo samples used to identify potential OXTR SNP variation of 7 missing founders. (DOCX) [file pone.0113364.s002.docx]

**Table S2. Individual information on age, sex and origin for additional bonobo samples used to identify potential *OXTR* SNP variation of 7 missing founders.**

| Studbook number | Sex | Year of Birth | Sire | Dam |
| --- | --- | --- | --- | --- |
| 134 | Female | 1985 | Bosondjo | Laura |
| 84 | Male | 1978 | Vernon | Catherine |
| 133 | Female | 1985 | Vernon | Loretta |
| 38 | Male | 1963 | Camillo | Margrit |
| 52 | Female | 1973 | Camillo | Margrit |
| 137 | Male | 1986 | Bono | Salonga |
| 132 | Female | 1985 | Bono | Natalie |
| 240 | Female | 1998 | Kidogo II | Hermien |
| 348 | Female | 2005 | Redy | Hermien |
| 376 | Male | 2007 | Ludwig | Natalie |
| 244 | Male | 1998 | Masikini | Lina |
| 164 | Male | 1991 | Mato | Lisala |
| 151 | Male | 1989 | Mato | Catherine |
| 386 | Male | 2008 | Keke | Maringa II |
| 159 | Male | 1990 | Mato | Lisala |
| 279 | Male | 2001 | Mato | Eja |
