# Supplementary material for: Oxytocin and Vasopressin Receptor Gene Variation as a Proximate Base for Inter- and Intraspecific Behavioral Differences in Bonobos and Chimpanzees
Source: PLoS One. 2014 Nov 18;9(11):e113364. doi: 10.1371/journal.pone.0113364 (PMC4236177; doi:10.1371/journal.pone.0113364)
Supplement: Table S3 — Individual information on age, sex, origin and genotype for chimpanzee samples used in this study. (DOCX) [file pone.0113364.s003.docx]

**Table S3. Individual information on age, sex, origin and genotype for chimpanzee samples used in this study.**

| Studbook number | Sex | Year of Birth | Sire | Dam | Genotype RS3 | NC_006490.3:g.8947139T>C |
| --- | --- | --- | --- | --- | --- | --- |
| 11051 | Female | 1966 | W | W | 140-140 | TT |
| / | Female | Unknown | Unknown | Unknown | 140-140 | TT |
| 11315 | Female | 1971 | W | W | 140-140 | TT |
| 10886 | Female | 1963 | W | W | 140-140 | TT |
| 11110 | Female | 1967 | W | W | 140-140 | CT |
| 11053 | Female | 1966 | W | W | 140-140 | TT |
| 11546 | Female | 1975 | 11134 | 11044 | 140-477 | TT |
| 11387 | Female | 1972 | 10493 | 10549 | 140-140 | TT |
| 11664 | Female | 1977 | Unknown | Unknown | 483-485 | TT |
| 11309 | Female | 1970 | 10885 | 10736 | 140-140 | TT |
| 11725 | Female | 1978 | Unknown | Unknown | 477-479 | TT |
| 10627 | Female | 1957 | W | W | 140-140 | TT |
| 11977 | Female | 1982 | 10936 | 10935 | 140-140 | CT |
| 12170 | Female | 1985 | Unknown | Unknown | 492-492 | TT |
| 10617 | Female | 1957 | W | W | 140-140 | CT |
| 11706 | Female | 1977 | 10413 | 10342 | 140-140 | TT |
| 11210 | Female | 1969 | W | W | 140-481 | TT |
| 11112 | Female | 1967 | W | W | 140-140 | CT |
| 11251 | Female | 1970 | W | W | 483-483 | TT |
| 11724 | Female | 1978 | Unknown | Unknown | 481-489 | TT |
| 12559 | Female | 1991 | Unknown | 10871 | 481-481 | TT |
| 11160 | Female | 1968 | W | W | 140-140 | TT |
| 10706 | Female | 1959 | W | W | 140-140 | TT |
| 10892 | Female | 1963 | W | W | 483-485 | TT |
| 11209 | Female | 1969 | W | W | 140-140 | TT |
| 11207 | Female | 1969 | W | W | 140-140 | TT |
| 10888 | Female | 1963 | W | W | 140-140 | TT |
| 12055 | Female | 1983 | 11132 | 11244 | 140-475 | TT |
| 12688 | Female | 1993 | 12082 | 11827 | 140-140 | TT |
| 12063 | Male | 1978 | Unknown | Unknown | 481-483 | TT |
| 12328 | Male | 1986 | Unknown | Unknown | 477-479 | TT |
| 11052 | Male | 1967 | W | W | 140-140 | TT |
| 12455 | Male | 1989 | 10662 | 10963 | 492-492 | TT |
| 10784 | Male | 1960 | W | W | 140-140 | CT |
| 11910 | Male | 1981 | W | W | 140-140 | CT |
| 10794 | Male | 1961 | W | W | 140-140 | CC |
